# Femoral Head Pathology in Subcapital Hip Fractures: Clinical Value and Cost-Effectiveness in a 230-Patient Case Series

**DOI:** 10.3390/diagnostics15020234

**Published:** 2025-01-20

**Authors:** Nissim Ohana, Omer Marom, David Segal, Refael Behrbalk, Yuval Ben-Sira, Alex Tavdi, Ezequiel Palmanovich, Eyal Yaacobi

**Affiliations:** 1 Orthopedic Department, Meir Medical Center, Kfar Saba, Faculty of Medical and Health Sciences, Tel-Aviv University, Tel Aviv 6997801, Israel; marom.omer@gmail.com (O.M.); dudisegal@gmail.com (D.S.); refael.behrbalk@yahoo.com (R.B.); ezepalm@gmail.com (E.P.); eyal.yaacobi@gmail.com (E.Y.); 2 Department of Orthopaedic Surgery, Meir Medical Center, 59 Tchernichovsky St., Kfar Saba 4428164, Israel; 3 Orthopedic Department, Shamir Medical Center (Assaf Harofeh), Faculty of Medical and Health Sciences, Tel-Aviv University, Tel Aviv 6997801, Israel; yuval.bensira@clalit.org.il

**Keywords:** subcapital fracture, femoral head, cost, pathology, malignancy

## Abstract

**Background/Objectives:** Osteoporotic fractures, particularly subcapital hip fractures (SCF), pose a significant healthcare and economic burden. The routine pathological examination of resected femoral heads in such cases is common practice, aimed at identifying malignancies that may have contributed to bone fragility. This study evaluated the cost-effectiveness and clinical utility of routine femoral head pathology in patients undergoing surgical treatment for SCF. **Methods:** A retrospective cohort study was conducted at a university-affiliated, tertiary care hospital. Patients undergoing surgical treatment for SCF between 2015 and 2018, with available femoral head pathology reports, were included. Data on demographics, prior or active malignancies, surgical procedures, and pathology results were analyzed. **Results:** The study included 230 patients with a mean age of 82.4 ± 14.1 years, of whom 57% were female. A total of 72 (31%) patients had a history of malignancy at the time of surgery. Pathological examination identified malignancies in eight patients (3.4%), all of whom had active malignancies at the time of admission. The most common malignancies detected were breast cancer and multiple myeloma (three cases each). None of the findings led to changes in patient management. **Conclusions:** The routine pathological examination of femoral heads following SCF provided a limited diagnostic yield and did not alter clinical management in this cohort. These findings suggest that routine pathology may not be cost-effective and support the adoption of selective screening approaches based on clinical risk factors such as a history of malignancy or atypical fracture presentations.

## 1. Introduction

Osteoporotic fractures, particularly proximal femoral fractures, represent a significant global healthcare and economic challenge [1]. These injuries result in substantial morbidity, heightened mortality, and reduced functional status, particularly among older adults [2]. In the United States alone, femoral neck fractures remain a significant healthcare concern among individuals aged 65 years and older, with the annual incidence declining from 242 per 100,000 in 2003 to 146 per 100,000 in 2013, as reported in a nationwide study analyzing geriatric trends [3]. Among these injuries, displaced intracapsular femoral neck fractures account for nearly one-third of cases, with treatment strategies tailored to patient functionality and overall health. Hemiarthroplasty is typically performed for individuals with limited physical activity and significant comorbidities, while total hip arthroplasty is reserved for those with greater self-sufficiency and activity levels [4].

Historically, such fractures were often classified as “pathological”, primarily due to their association with decreased bone quality resulting from osteoporosis and the low-energy mechanisms involved [4,5,6]. This perspective led to the widespread practice of the routine pathological examination of resected femoral head specimens. These examinations aim to identify abnormalities such as osteoporosis, primary malignancies, metastases, avascular necrosis, infection, or trauma, offering potential insights into the etiology of the fracture. However, the utility of these routine assessments remains controversial [7]. Questions persist regarding whether the findings from femoral head pathology examinations lead to changes in patient management that justify their costs and resource utilization.

Routine histological examination following non-oncological surgeries has long been a standard practice, aimed at identifying unexpected pathologies that may influence postoperative management [8,9]. However, its necessity and cost-effectiveness have been increasingly questioned. For example, in routine appendectomies, histological evaluation occasionally identifies unexpected malignancies such as carcinoid tumors, but these findings are rare and often associated with macroscopically suspicious features [10]. Similarly, in cholecystectomy specimens excised for benign gallbladder diseases, routine histology has been shown to detect incidental gallbladder cancers in a small fraction of cases (e.g., 0.4%), yet these findings frequently align with preoperative or intraoperative suspicions [10,11]. In lumbar and cervical discectomies, routine histopathological evaluation rarely uncovers clinically significant diagnoses and has not been shown to alter management in cases of benign degenerative conditions [12]. These studies collectively emphasize that while routine histology provides a safety net, its low yield in detecting unexpected pathologies in non-oncological surgeries highlights the potential for more selective approaches. By reserving histological examination for cases with clinical or intraoperative indicators of malignancy or infection, healthcare systems can optimize resource allocation without compromising patient care.

The increasing life expectancy and the growing elderly population predict a sustained rise in the incidence of osteoporotic fractures, particularly those involving the hip [13]. This trend amplifies the urgency of addressing cost containment while maintaining effective care. Many healthcare institutions implement the routine pathological examination of resected femoral heads as part of standard practice, although this approach varies by institution and is not universally adopted [7,10]. These protocols often operate without regard to patient-specific factors such as age, gender, or medical history [14,15,16]. While this approach ensures comprehensive evaluation, it raises questions about its clinical value and cost-effectiveness, particularly in the context of resource-intensive healthcare systems.

The purpose of this study is to evaluate the utility of femoral head pathology examinations as a screening tool for bone malignancies. While similar previous investigations have largely focused on the cost-effectiveness of routine pathological evaluations [7], others have examined routine pathology for knee arthroscopy specimens, finding that discordant diagnoses were exceedingly rare (0.026%) and associated with an estimated cost of USD 371,810 per discordant diagnosis, raising questions about its cost-effectiveness [17]. Our study aims to explore whether specific patient populations or clinical scenarios might benefit from targeted pathological screening. By examining the diagnostic yield and potential clinical implications of femoral head pathology, we seek to determine whether a more selective approach could optimize its value in orthopedic and oncological practice.

## 2. Methods

### 2.1. Study Setting and Population

We conducted a retrospective cohort study, classified as level 3 evidence, following approval from our institutional review board (265-20-MMC, approved on 3 December 2020). The study adhered to the ethical principles outlined in the Declaration of Helsinki, as revised in October 2024.

The research was carried out at a university-affiliated, tertiary care hospital and included all patients who underwent surgical treatment for subcapital fractures (SCF) between 1 January 2015 and 31 December 2018. Patients without femoral head postoperative pathology analysis were excluded from the study. SCF were diagnosed by specialized orthopedic surgeons, each with a minimum of 8 years of expertise, based on clinical examinations and imaging studies.

**Figure 1:**
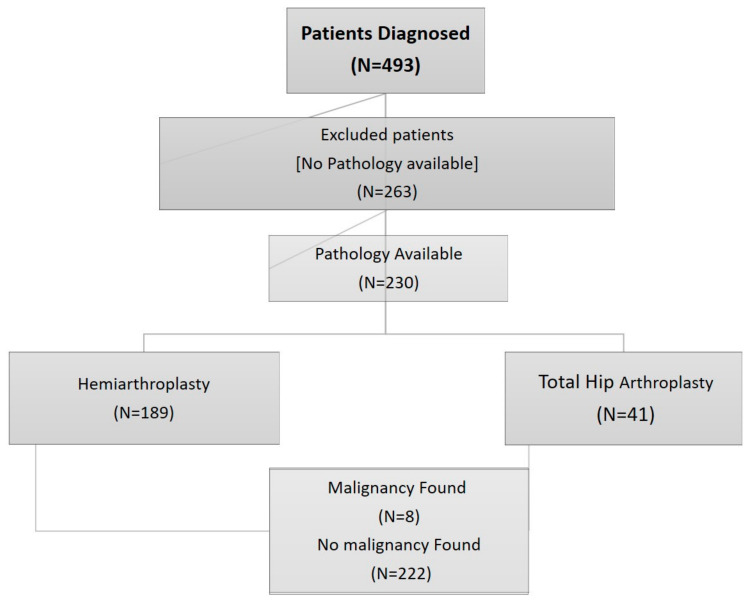
Flowchart of patient selection and analysis. A total of 493 patients underwent surgical treatment for subcapital fractures. Of these, 263 were excluded due to the absence of femoral head pathological examination, leaving 230 patients for analysis.

The type of surgical intervention was determined according to the fracture type and the patient’s medical and functional status. Patients with limited physical capabilities typically underwent partial hip arthroplasty, while those with higher levels of physical activity were treated with total hip arthroplasty. During the surgery, the attending orthopedic surgeon determined the type of implant to be used—cemented or non-cemented—based on the stability of the implant. This decision subsequently dictated whether bone cement was required during the procedure. Following excision, all femoral heads were sent to the pathology laboratory for further examination. Particular emphasis was placed on identifying evidence of malignancy, given the potential impact on postoperative management and treatment (Figure 1).

### 2.2. Laboratory Measurements and Data Collection

The data collected from electronic medical records included patients’ demographic characteristics, background diagnosis at the time of admission, data on the procedure performed, femoral head pathology reports, and postoperative follow-up and complications. The collection of data was approved by the Institutional Review Board. During the study period, fractured femoral heads, naturally detached due to SCF, were routinely analyzed in our medical facility’s pathology laboratory. Full histological and pathological examinations were performed to detect abnormalities. According to the pathology lab protocol, the articular surface was first examined for irregularity, osteophyte lipping, and synovial membranes, either heterophilic or papillary. An incision was then made in the center of the articular surface, followed by a second incision, creating a specimen about 3 mm thick. The excised slice of the femoral head was then examined for the thickness of the articular cartilage, subchondral eburnation or cysts, areas of necrosis, malignancy, and evidence of fracture. Subsequently, two sections from the most abnormal areas, at least one including the articular surface and synovium, were fixated in formalin for several hours or overnight to decalcify thoroughly. Further testing was conducted based on the histopathologic lesion in cases where pathology indicated a positive result, including immunohistochemical (IHC) staining to detect specific tumor markers and molecular diagnostic tests, such as gene expression profiling, to identify the primary disease.

### 2.3. Statistical Analysis

Data were collected and analyzed with SPSS 27.0 software (Chicago, IL, USA). Descriptive statistics were used to present raw data. We planned to conduct a univariate comparison that would be followed by a multivariable logistic regression to characterize the subgroup of patients who would benefit from a pathological test, but this was deemed unnecessary in light of the results.

## 3. Results

**Figure 2:**
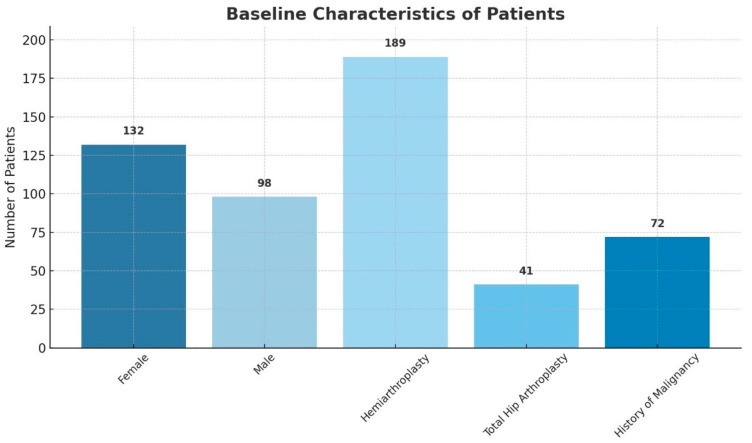
This figure illustrates the baseline characteristics of the study population, categorized by gender, treatment type, and history of malignancy.

During the study period, 493 patients underwent surgical treatment for subcapital fractures (SCF) at our medical center. Of these, 263 patients (53%) were excluded from the analysis due to the absence of a pathology examination of the femoral head. The remaining 230 patients were included in the study, with treatment distributed between total hip arthroplasty and partial hip arthroplasty, as detailed later in the text. Figure 2 illustrates the characteristics of the remaining 230 patients included in the study cohort.

### Baseline Characteristics

The mean age of the 230 patients in the cohort was 82.4 ± 14.1 years; 132 (57%) were women. A total of 41 (18%) underwent total hip arthroplasty and 189 (82%) underwent partial hip arthroplasty. For 72 (31%) patients, their medical history included past or active malignancy at the time of the operation (Figure 2).

**Figure 3:**
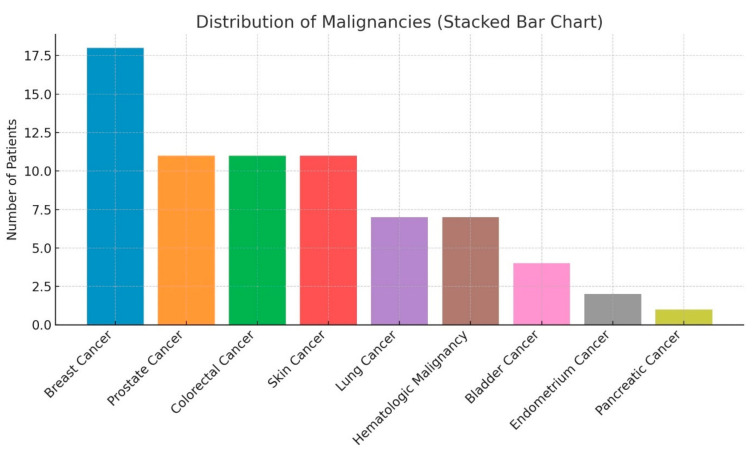
The bar chart illustrates the distribution of malignancies among patients with a history of cancer, categorized by cancer type.

Figure 3 shows that breast cancer was the most common malignancy, accounting for 25% of cases (18 patients). Prostate, colorectal, and skin cancers each contributed 15% (11 patients). Lung cancer and hematologic malignancies comprised 10% of cases (7 patients each), while bladder cancer represented 5% (4 patients). Less common malignancies included endometrial cancer (3%, 2 patients) and pancreatic cancer (1%, 1 patient).

**Figure 4:**
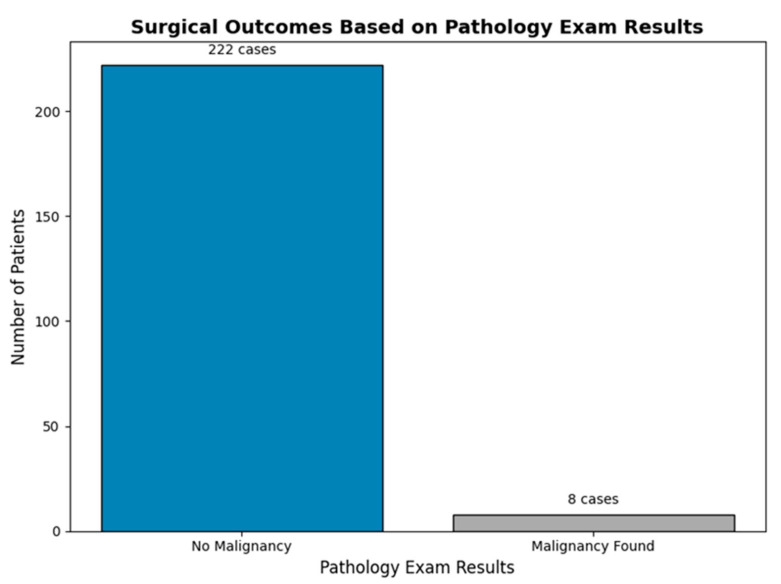
Distribution of pathology results among 230 patients, showing cases with and without malignancy identified through femoral head examination.

Of the 230 patients included in the study, 8 (3.4%) were found to have malignancies identified through femoral head pathology examination, while 222 (96.6%) showed no evidence of malignancy (Figure 4).

**Figure 5:**
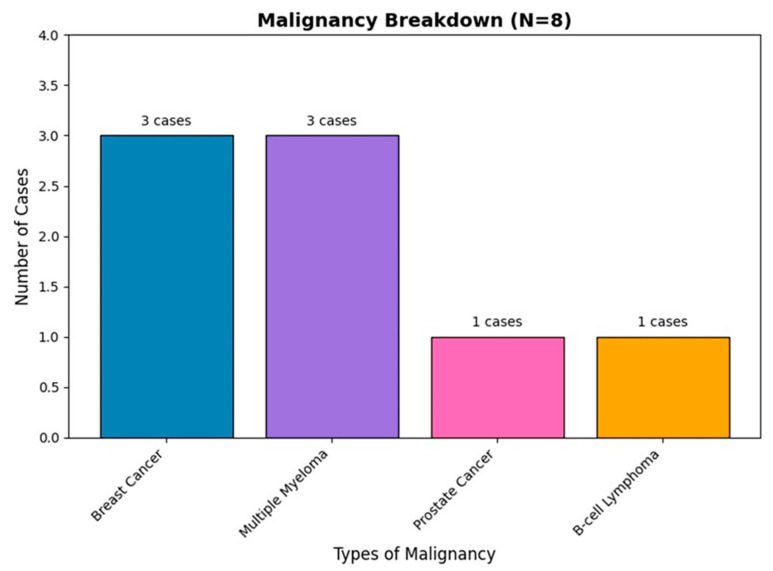
Breakdown of malignancy types identified (N = 8). The chart displays the distribution of malignancy types among patients, categorized by cancer type.

All 8 patients with malignancies had current, active cancer at the time of the fracture and operation. The most common malignancies identified were breast cancer and multiple myeloma, each found in three patients (38%), while prostate cancer and B-cell lymphoma were identified in one patient each (Figure 5).

Among the eight patients with malignancies, five (63%) reported experiencing proximal hip pain several months prior to the diagnosis of the fracture, suggesting possible underlying pathological changes. For the remaining three patients, direct trauma to the proximal hip area resulted in fractures following a fall. All eight patients were already undergoing treatment for active cancer at the time of the fracture; therefore, no changes were made to their diagnosis or treatment plans as a result of the pathology findings.

## 4. Discussion

The main finding of this study is that pathological examinations of the removed femoral head did not alter the medical management of any of the 230 patients analyzed for SCF. This indicates that the level of suspicion for unknown tumors in the femoral head, which might have contributed to the fragility of this predominantly weak anatomic site, should be considered low. Consequently, the number-needed-to-test to yield clinically meaningful findings exceeds the current study population (n = 230). These results are consistent with previous investigations, including findings from a systematic review [7], which analyzed 17,388 femoral head specimens retrieved during total hip arthroplasty. That study reported that pathological diagnoses influenced patient management in only 0.0058% of cases, further emphasizing the limited clinical utility of routine femoral head pathology. Additionally, the study highlighted the significant economic burden associated with this practice, estimating an annual cost of up to USD 63 million in the United States alone. These findings collectively reinforce the argument that the routine pathological examination of femoral heads provides limited utility and should be reconsidered in favor of selective, risk-based approaches [18,19,20,21,22,23]. Routine histological examination in non-oncological surgeries has been the subject of ongoing debate regarding its necessity and cost-effectiveness. Matthyssens et al. [10] demonstrated that routine pathology in procedures such as appendectomy, cholecystectomy, and hernia repair rarely identified findings of clinical significance. For example, among over 1465 appendectomy specimens analyzed, only 0.1% revealed an incidental carcinoid tumor, which was neither macroscopically nor clinically suspected. Similarly, their analysis of 1523 cholecystectomy specimens found that all cases of gallbladder carcinoma were macroscopically apparent, underscoring the limited value of routine histology in specimens without gross abnormalities [11]. These findings support broader recommendations advocating for selective histopathological examinations, particularly when preoperative or intraoperative observations suggest potential pathology. This perspective is highly relevant to femoral head pathology, where routine examination has demonstrated a similarly limited impact on patient management, further emphasizing the need for selective, risk-based screening approaches [7].

Davis et al. also reported that pathology examinations of femoral head specimens did not change the management of any of the 466 patients analyzed [24]. Unlike the current study, in which less than half of the resected femoral heads were sent for pathological examination, Davis et al.’s study systematically included only 54% of the specimens due to non-consecutive patient selection. Despite this methodological difference, the conclusion regarding the minimal clinical value of routine femoral head pathology remains consistent across studies.

Proximal femoral fractures have been described as among the most expensive fractures to treat [25]. This is due to both the total costs and the growing elderly population [18]. A cost-effectiveness evaluation of the various components of the treatment can save a substantial amount of capital, which can be allocated elsewhere and more effectively. Together with previous publications, the current study should prompt medical centers to conduct hospital-specific cost-effectiveness evaluations to facilitate surgeons and medical organizations in planning the utility of pathology services for femoral fractures in older patients.

At our institution, the cost of a single pathology examination of a femoral head is USD 400. Given that no positive findings were detected in this study, the cost of identifying a single positive case is effectively greater than USD 92,000 (USD 400 multiplied by 230 patients). For comparison, mammography screening costs USD 86–137 per test [26], with a reported number-needed-to-test of 746–1316 to prevent one death from breast cancer, at a cost of USD 64,000–181,000 [27]. Colonoscopy screening, by contrast, costs USD 800–1000 per test [28], with a number-needed-to-test of 1250 patients per disease detection, amounting to costs exceeding USD 992,500.

However, as with any screening method, cost-effectiveness depends on the diagnostic yield and clinical relevance. In this study, the 3.4% malignancy detection rate identified only known cases and did not alter management [29].

Additional considerations, such as the costs of late cancer detection, treatment expenses, and quality-adjusted life years saved, should also inform the evaluation of screening tests. For instance, the early detection of colorectal polyps can prevent late-stage colorectal cancer, saving both lives and substantial healthcare costs [28]. Conversely, detecting bone metastases in femoral heads typically identifies tumors that are already symptomatic and advanced, offering fewer life years and limited economic benefit [29]. While a detailed value analysis is beyond the scope of this study, comparisons with other screening tests provide a useful context for evaluating femoral head pathology.

Among patients with a history of malignancy, eight cases of malignancy were identified, while no malignancies were detected in patients without such a history. This underscores the importance of targeted screening based on clinical risk factors such as cancer history, unexplained proximal hip pain, or fractures occurring without significant trauma. Persistent, unexplained pain is a particularly important clinical indicator. Gheita et al. [30] emphasized that bone pain, often worsening at night and unresponsive to standard analgesics, is a hallmark of metastatic bone disease and should prompt further investigation. Incorporating such symptoms into screening criteria could improve the diagnostic yield of femoral head pathology, prioritizing high-risk cases and enhancing cost-effectiveness.

Selective screening should also consider tumor types commonly known to metastasize to bone, including lung, breast, prostate, colorectal, thyroid, gynecologic, hematologic, and melanoma malignancies [29]. Paraneoplastic symptoms, such as weight loss, fatigue, or localized pain, should further guide the decision to perform pathology [31]. By integrating these criteria, hospitals and healthcare providers can better balance the costs and benefits of femoral head pathology.

In contrast to the limited utility of routine pathological examinations, microbial evaluations of surgical specimens, such as heart valves [32] and joint fluid prior to revision arthroplasty [33], often yield clinically actionable information. For instance, microbial cultures from heart valves can detect silent endocarditis, while joint fluid cultures in revision arthroplasty can identify low-grade infections, both of which significantly influence postoperative management. These examples underscore the importance of tailored approaches to diagnostic testing, reserving routine evaluations for cases with clear clinical indications.

## 5. Clinical Lessons

The findings of this study highlight several important clinical lessons.

1. The routine pathological examination of femoral heads in SCF has limited value and should be reserved for cases with clear clinical indications, such as a known history of malignancy or symptoms suggestive of bone metastases.
2. Persistent proximal hip pain in patients with an existing or prior malignancy, especially if unexplained or resistant to standard treatment, should prompt further diagnostic evaluation, as it may be a critical clue to underlying malignancy contributing to the fracture.
3. The integration of selective pathology into clinical workflows, particularly for patients with a history of malignancy or atypical fracture presentations, may optimize resource allocation. Although this study does not directly assess resource efficiency, the results highlight the limited utility of routine pathology in the absence of specific risk factors, suggesting that a targeted approach could enhance diagnostic efficiency.
4. While broader recommendations regarding pathology services in orthopedic practice are supported by prior studies, the findings of this study specifically support the development of institutional guidelines that prioritize selective pathology for high-risk patients. This approach aligns with the observed limited utility of routine examinations in a general patient population.

## 6. Limitations

This study has several limitations:

1. Retrospective Design: The retrospective nature of the study introduces inherent challenges, including potential selection bias and incomplete data collection.
2. Sample Size: The limited patient population did not meet the minimum number-needed-to-test to draw definitive conclusions about the utility of routine femoral head pathology in detecting malignancies.
3. Single-Center Study: The study was conducted at a single medical center serving a specific local population. This may limit the generalizability of the findings to other populations with differing demographic, clinical, or epidemiological characteristics.

## 7. Conclusions

This study demonstrates that the routine pathological examination of femoral heads following SCF has limited diagnostic utility and does not significantly impact patient management. The findings align with prior research indicating the minimal value of routine pathology in non-oncological surgical contexts. Selective screening based on specific risk factors, such as a history of malignancy, atypical fracture presentation, or tumor-related symptoms, offers a more targeted and cost-effective approach.

Tailoring pathology services to clinical scenarios based on these findings can optimize resource allocation and improve patient care. Multi-center prospective studies could further validate these findings and refine guidelines for the selective use of pathology services in orthopedic practice.

## Data Availability

The data presented in this study are available on request from the corresponding author. The data are not publicly available due to ethical and privacy restrictions.
